# Effects of Occlusal Splints on Spinal Posture in Patients with Temporomandibular Disorders: A Systematic Review

**DOI:** 10.3390/healthcare10040739

**Published:** 2022-04-15

**Authors:** Martina Ferrillo, Nicola Marotta, Amerigo Giudice, Dario Calafiore, Claudio Curci, Leonzio Fortunato, Antonio Ammendolia, Alessandro de Sire

**Affiliations:** 1 Dentistry Unit, Department of Health Sciences, University of Catanzaro “Magna Graecia”, 88100 Catanzaro, Italy; martinaferrillo@hotmail.it (M.F.); a.giudice@unicz.it (A.G.); leo@unicz.it (L.F.); 2 Physical Medicine and Rehabilitation Unit, Department of Medical and Surgical Sciences, University of Catanzaro “Magna Graecia”, 88100 Catanzaro, Italy; ammendolia@unicz.it (A.A.); alessandro.desire@unicz.it (A.d.S.); 3 Physical Medicine and Rehabilitation Unit, Department of Neurosciences, ASST Carlo Poma, 46100 Mantova, Italy; dario.calafiore@asst-mantova.it (D.C.); claudio.curci@asst-mantova.it (C.C.)

**Keywords:** temporomandibular disorders, temporomandibular joint disorders, body posture, posture, stabilometry, occlusal splints, splints, stabilization splints

## Abstract

There is still a gap in the scientific knowledge on the linkage between craniofacial structure and spinal postural control in temporomandibular disorder (TMD) patients. This systematic review aimed to assess the role of occlusal splints on spinal posture of TMD patients. PubMed, Web of Science, and Scopus were systematically searched from inception until 5 January 2022 to identify observational studies with a longitudinal study design presenting: patients with diagnosis of TMD according to the Diagnostic Criteria for Temporomandibular Disorders (DC/TMD); occlusal splint therapy as intervention; postural assessment as outcome. Out of 133 records identified, 104 were suitable for data screening, and only 7 articles were included satisfying the eligibility criteria. We found that occlusal splints might have a positive effect on posture in TMD patients, albeit there is little evidence of appropriate investigation for postural assessment. This systematic review suggested that the occlusal splint might be considered a non-invasive therapeutic approach for patients with TMD. However, the low number of studies with high-quality methodology in these patients showed an urgent need for further research using combined force platform stabilometry and kinematic evaluation of the spine to investigate the impact of occlusal splints on posture.

## 1. Introduction

Temporomandibular disorder (TMD) is a collective term, referring to a large number of musculoskeletal disorders involving the masticatory muscles, the temporomandibular joint (TMJ), and the surrounding structures [1]. According to the Diagnostic Criteria for Temporomandibular Disorders (DC/TMD) [2] Axis I, TMD could be classified into three groups: Group I, muscular disorders; Group II, disc displacement; and Group III, arthralgia and arthritis [2]. TMD might also be related to a prolonged use of mastication muscles, grinding and clenching (as parafunctional activities), repetitive trauma at the TMJ, psychological disorders (e.g., anxiety and depressive syndrome), and cervical posture [3]. Albeit TMD management is debated in literature, to date, the first-line approaches are still considered the conservative and rehabilitative therapies, including physical therapy, orthodontics, oral non-steroidal anti-inflammatory drugs, electrotherapy, occlusal splints, extracorporeal shockwave therapy, oxygen-ozone therapy, and occlusal splints [4,5,6,7,8,9,10,11,12,13,14,15].

In this scenario, the occlusal splints seem to play a key role and are commonly used for TMD-related pain relief [11,12,13,14,15]. More in detail, there are several types of occlusal splints (e.g., bite plates) with different indications and functions. The stabilization splints are hard acrylic devices that provide a temporary and removable ideal occlusion (adequate contact between the upper and lower arches) for TMD patients, thus reducing orofacial pain by relaxing masticatory muscles [13,14,15,16,17,18]. The stabilizations splints might lead to a neuromuscular balance, eliminating the posterior interferences and providing a centric relation occlusion and stable occlusal relationship [19,20,21].

In this context, TMJ is connected to the cervical region via muscles and ligaments; the cranium, mandible, and cervical spine present neurological and biomechanical interactions, forming a functional complex that might be defined as “craniocervical mandibular system” [22]. To date, the potential correlation between craniofacial morphology and posture of the spinal column is still considered a controversial issue in the scientific literature [23].

Therefore, TMD are craniocervical mandibular disorders, which could produce moderate postural instability even in patients with a normal vestibular function [24,25]. Indeed, head and neck posture might be considered as an indicator of the biomechanical equilibrium of the cranium and upper cervical spine, considering that TMD and cervical spine disorders share pathophysiological mechanisms [26,27]. Furthermore, patients with cervical myofascial pain may present antalgic forward head posture to reduce pain by shortening the vertical extensor muscle, thus potentially developing trigger points in cervical and masticatory muscles [28,29,30,31].

Posture is comprehended as the position of the human body and its orientation in space [32,33]. It implicates neuro-muscular activation governed by the central nervous system, which permits postural adjustments as a result of a convoluted mechanical system stimulated by integrated, multisensory inputs [34,35,36]. Disturbances of this scenario are frequently observed in people who complain of dizziness, being one of the most common symptoms either in otologic or neurological clinics [35,37]. The most frequently used strategy to study postural control consists of evaluating the oscillation of the body during erect and resting posture employing a force platform [38,39].

An adequate maintenance of body posture is a set of interactions between the muscle–skeletal system with afferent and efferent pathways of the central nervous system, which allow a safe execution of most movements [40,41,42]. It has recently been supposed that there is a relationship between the stomatognathic system and spinal posture control, thus making a deeper understanding of this linkage necessary [22,43,44]. However, it should be taken into consideration that the correlation between spine posture and craniofacial morphology might be influenced by skeletal and dental maturation [45,46,47,48,49].

Considering the gap in scientific knowledge on this topic, we believe that it is necessary to better understand the linkage between craniofacial structure and spinal posture in patients with TMD.

Therefore, the aim of this systematic review was to investigate the current literature on the effects of occlusal splints on spine posture in patients affected by TMD, to contribute to the knowledge of this complex relationship between TMD management and posture.

## 2. Materials and Methods

### 2.1. Search Strategy

A Technical Expert Panel was established by physicians with expertise in physical and rehabilitative medicine (PRM) and dentistry, involved in the management of TMD patients. This panel, including all the authors, defined the objective of this systematic review and proposed the search strategy. PubMed, Scopus, and Web of Science databases were systematically searched for articles published from the inception until 5 January 2022, according to each specific thesaurus, following the strategy described by Table 1.

Furthermore, a manual search of the references of previous systematic reviews on similar topics was conducted as well.

This systematic review with meta-analysis was conducted according to the guidance of Preferred Reporting Items for Systematic Reviews and Meta-Analyses (PRISMA) guidelines [50] and the Cochrane Handbook for Systematic Reviews of Interventions [51]. The systematic review protocol was registered on the International Prospective Register of Systematic Reviews (PROSPERO) with number: CRD42022303280.

### 2.2. Selection Criteria

Two reviewers (MF, NM) independently screened all potential papers for eligibility after duplication removal. Any disagreement has been resolved through discussion or, if necessary, by a consultation of a third reviewer (AdS).

All studies were assessed for eligibility according to the following PICO model:

(P) Participants consisted of patients with diagnosis of TMD;

(I) Intervention consisted of occlusal splints;

(C) Comparator: not applicable;

(O) Outcome measure consisted of postural assessment.

We included observational studies with a longitudinal study design. We excluded: (1) studies on children; (2) patients with fibromyalgia; (3) patients with headache/migraine; (4) studies written in a language different from English; (5) full-text unavailability (i.e., posters and conference abstracts); (6) studies involving animals.

### 2.3. Data Extraction

Two reviewers (MF, NM) independently extracted data from included studies using a customized data extraction on a Microsoft Excel sheet. In case of disagreement, the consensus was achieved through a third reviewer (AdS). A descriptive approach was used to synthesize both study characteristics and data extracted. The following data were extracted: first author, journal, publication year, nationality, study design, population and number of patients included, age of study participants, type of occlusal splint, postural assessment as outcome, and main findings.

### 2.4. Data Synthesis

We adopted the risk-of-bias checklist in Joanna Briggs Institute Critical Appraisal Checklist for Quasi-Experimental Studies (non-randomized experimental studies) [52] to estimate the included studies’ methodological quality. Two authors (MF, NM) separately evaluated each article and presented the results, and any disagreements were resolved involving a third author (AdS). The JBI-QES tool consists of nine domains through which it is possible to find any bias in a study. Options for each judgment are low risk of bias, moderate risk of bias/some concerns, serious risk of bias, critical risk of bias, and no information. Domain-level reports provide the basis for an overall risk-of-bias judgment.

## 3. Results

### 3.1. Main Characteristics of the Included Studies

A total of 133 records were identified from the search process; after the duplication removal, 104 papers were screened for title and abstract and 86 of them were excluded. Then, out of the 18 full-text studies screened, 7 articles [53,54,55,56,57,58,59] were included that satisfied the eligibility criteria. Table 2 describes the reasons for article exclusion by the present systematic review.

**Figure 1:**
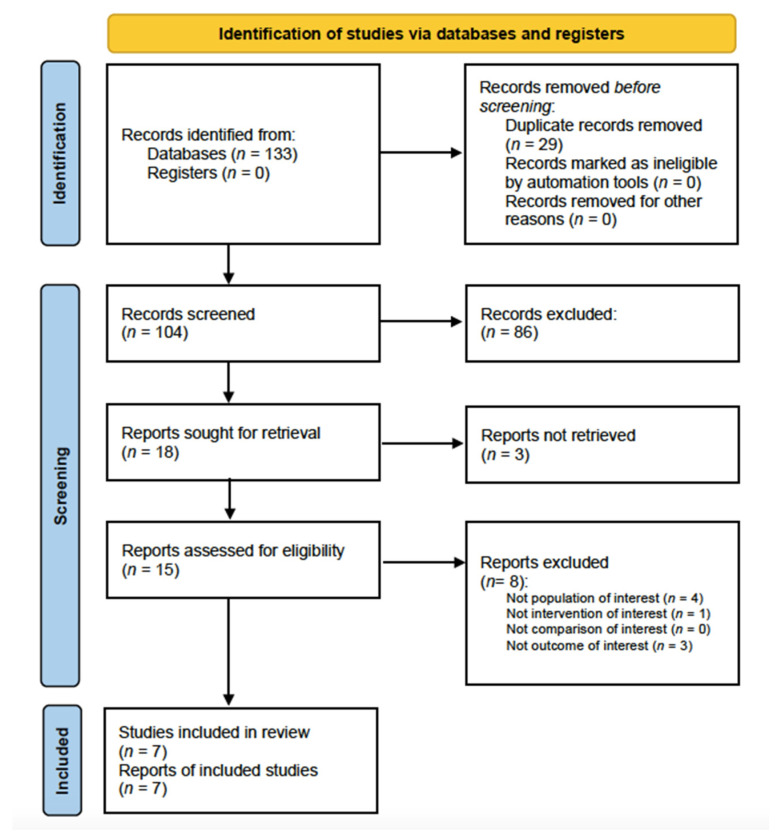
PRISMA 2020 Flow diagram.

Further details on the identification and inclusion/exclusion of the screened studies are reported in the PRISMA 2020 Flow diagram (Figure 1).

These studies were published between 2009 and 2021. Most of them were from Brazilian and Italian collaborations (both 28.5% of the manuscripts, respectively); 3 studies were carried out in Europe [54,55,56], 2 in South America ([53,57]), 1 in Korea [58], and 1 in Lebanon [59]. The number of patients included ranged from 1 (a case report study design) [54] to 187 subjects [58].

We included four observational studies [53,55,58,59], two RCT [56,57] and one case report [54]. The spinal posture was mostly assessed through stabilometry on a force platform (*n* = 3) [54,57,59]; the other papers evaluated posture using other techniques: head position assessment (*n* = 1) [53], computerized cephalometric analysis (*n* = 1) [58], kinematics of the cervical spine (*n* = 1) [55], and rasterstereography (*n* = 1) [56]. Main characteristics of the seven papers included [53,54,55,56,57,58,59] are summarized in Table 3.

### 3.2. Occlusal Splints

Strini et al. provided [53] an upper arch occlusal splint fabricated by programming the mandibular position in centric relation occlusion, occlusal stability, and anterior guidance for each patient. The occlusal splint was adjusted according to the occlusal forces directed to the long axis of the posterior teeth, over their occlusal faces, eliminating any contact between upper and lower anterior teeth. Moreover, the patients were instructed to use the splint continuously for 24 h within the first week of treatment, and after this period, to use the splint every night, being free to use it during the day in case of pain. With a case report, Baldini et al. [54] reported a resin stabilization splint, designed for the pilot’s lower arch, allowing for the unobstructed excursive glides of the mandible in the protrusive position, and laterality and occlusal balance in the centric position. It was designed to allow him to wear it in flight, maintaining the mandibular position in centric relation occlusion. Walczynska-Dragon et al. [55] supplied patients with an occlusal splint SVED (Sagittal Vertical Extrusion Device). This appliance made contact only with the anterior teeth in the opposing arch, disengaging the posterior teeth and thus eliminating their influence in the function of the masticatory system. Patients were ordered to wear the occlusal splint during sleep, but not more than 8–10 h per day. De Giorgi et al. [56] proposed a hard, acrylic resin stabilization splint made for the mandibular arch of each patient, applying a 2 mm thick device only for posterior contacts (from the second premolar to the second⁄first permanent molar), without static and dynamic anterior contacts. Patients wore the occlusal splint all night. Oliveira et al. [57] assessed the use of an occlusal splint with simultaneous bilateral contacts with absence of interferences in canine and anterior guides. This appliance allowed them to place the mandible in centric relation and to create simultaneous bilateral contacts, canine guide, and anterior guide in the occlusal splint. The occlusal splint was used throughout the night plus 2 h in the morning and 2 h in the afternoon. In Kang et al. [58], a stabilization splint was fabricated in an acrylic resin of 2 mm thickness for the molar area, covering maxillary teeth and creating uniform contact points for the lower functional cusps against the stabilization splint on the occluding premolar and molar teeth. All patients were instructed to wear the stabilization splint every night for at least eight hours per day. Lastly, El Zoghbi et al. [59] assessed the use of an occlusal splint fabricated for the upper arch, which guided the mandible to the centric occlusion to allow for maximum contact. The patients were advised to wear it during the night.

### 3.3. Posture Analysis

Baldini et al. [54] showed a good postural balance with a force platform, both with and without the occlusal splint. Nonetheless, a significant reduction in the sway index, the oscillation area of the body’s center of gravity could be achieved with the occlusal splint. Oliveira et al. [57] added to analyses of the center of pressure (COP) with the adjustment of visual input. Therefore, patients of the test group presented a significant increase in antero-posterior velocity from the COP with eyes open (*p* = 0.023) and eyes closed (*p* < 0.001); the control group did not present a significant increase in the eyes open (*p* = 0.249) primary outcome only with eyes closed (*p* < 0.046). El Zoghbi et al. [59] stated that the mean sway length might decrease significantly after the placement of the occlusal guard when participants were in a lateral position with open eyes (*p* = 0.025). Although the length decreased after placing the occlusal, the difference was not statistically significant in a static position with closed or open eyes, neither for the lateral position with closed eyes nor for the anteroposterior position with closed eyes.

Strini et al. [53] assessed the position of the head by placing a millimeter ruler vertically from the posterior portion of the occiput to the thoracic spine. Since then, the perpendicular distance of the cervical spine from that vertical weight line has also been measured with a centimeter ruler (with a normal distance ranging from 6 to 8 cm). Kang et al. [58] analyzed craniofacial features and head and neck posture by cephalometric analysis using V-ceph^®^ 5.0 software (Cybermed, Seoul, Korea). On the contrary, Walczynska-Dragon et al. 2014 [55] used an ultrasonic-based device (Zebris, GmbH, Isny im Allgäu, Germany) to collect external kinematic analysis of the cervical spine movements. Patients with a neutral (comfortably seated) position performed maximal head movements: flexion, extension, rotation to the right and left side, and lateral flexion movements. Finally, De Giorgi et al. [56] has set a rasterstereography to obtain a radiation-free representation of the patient’s dorsal profile.

### 3.4. Risk-of-Bias Analysis

To evaluate the quality of evidence included in this review, we adopted the Joanna Briggs Institute Critical Appraisal Checklist for Quasi-Experimental Studies (non-randomized experimental studies) [52]. As depicted in Table 4, we assessed the 9-question risk-of-bias domains.

All included articles had full-text availability. All studies were judged with at least one serious risk of bias, which translated into an overall serious risk of bias for that study. Included studies reported a lack of data on baseline characteristics of participants, non-random sampling approaches (convenience samples), missing data, and lack of a reliable tool to estimate and report outcomes.

## 4. Discussion

This systematic review investigated the role of occlusal splints on spine posture in patients with TMD, reporting a good postural impact with the use of these devices, without underestimating the different interventions and analyses, as well as the presence of only three controlled studies [55,56,57] out of seven included [53,54,55,56,57,58,59].

As reported by our results, there is a high heterogeneity in terms of occlusal splint intervention (time during day, duration of intervention, and practical applications). Indeed, most of the TMD patients in the included studies wore the occlusal splints during the night or for a minimum of 8 h per day.

A correct posture is particularly associated with health status and any alteration of this posture could lead to pain and dysfunction of the craniocervical system [60,61,62]. As treatment of TMD, occlusal splint might be effective in muscle relaxation, reducing hyperactivity of the masticatory muscles [63]. In this context, El Zoghbi et al. [59] supported the hypothesis that the stomatognathic system and postural body balance could be intimately interrelated; thus, the authors suggested that spine posture assessment could be considered as a key parameter in the management of TMD, starting from the posture control of the mandible by occlusal splint and body posture in TMD patients.

In a recent systematic review, Szczygieł et al. [64] aimed to assess the effects of an incorrect head position on the functioning of the human body. Results confirmed that changes of the head position led to accessory muscle recruitment with increased, among others, sternocleidomastoid and anterior scalene muscle activity causing rib cage elevation and reducing thoracoabdominal mobility, concluding that this series of events would be cervical spine overload syndromes [65,66].

It was postulated that modifications of the occlusal balance could resonate with the postural balance by adapting the morphostatic pattern of the subject, and proprioceptive distribution of the muscular tonic balance [59]. Therefore, the orofacial pain management in patients with TMD might show improvement of the neck pain and posture.

In this scenario, the effect of the use of the occlusal splints reported an increase in the speed of movement of the COP in the antero-posterior direction with open and closed eyes without a corresponding increase in the amplitude of the swing; Oliveira et al. [57] interpreted this phenomenon as an attempt of postural readjustment. The increase in the speed of postural oscillations might be considered as an increase in the frequency of corrections in the control of postural balance, triggered by the realignment of the head and neck posture [54].

On the other hand, the use of clinical and instrumental approaches for assessing body posture is not supported by the wide majority of the existing literature, mainly because of wide variations in the measurable variables of posture [67]. Particularly, the evidence did not support the usefulness of posturography as a diagnostic aid in dentistry because these analysis systems did not add significant advances [68]. In this regard, it becomes less clear how to instrumentally understand the connection between malocclusion and postural control. Indeed, only one study [55] has provided a functional assessment of the spine, with a kinematic analysis using an ultrasound device. Walczynska-Dragon et al. [55] reported a correlation between the pathologies and the positive impact of treatment within the motor aspect of the stomatognathic system on the alleviation of spine pain, even in subjects experiencing such pain for many years. However, the study was not a randomized controlled trial and the increase in the cervical spine range of motion might not necessarily lead to a functional improvement in the posture of TMD patients.

There was a low methodological quality in all the included studies. However, the lack of standardized consensus on the diagnostic-functional assessment of the posture of these patients has led to large numbers of inconsistent outcome measures. Thus, a quantitative meta-analysis was not possible. Furthermore, there is also a high heterogeneity in the TMD diagnosis in the included studies (myogenous, arthrogenous, and mixed TMD) that might affect the data. A larger sample study included only female patients with TMD, and on the other hand, Kim et al. [69] found sex differences between TMD patients in terms of quality of life and TMD symptoms. Furthermore, an epidemiological study revealed that female patients with TMD seek treatment more frequently than male patients with TMD [70,71].

However, it should be noted that, to the best of our knowledge, this is the first systematic review that investigated the role of occlusal splints on spine posture in patients with TMD.

## 5. Conclusions

Given the results, we might conclude that there is a consensus in the scientific literature on the use of rigid occlusal splints with a flat occlusal plane as a non-invasive therapeutic approach for patients with TMD. However, the clinical significance of the effects on postural parameters is still poor. A multidisciplinary approach involving PRM and dentistry specialists is mandatory for adequate diagnosis and management of posture in TMD patients. Further high-quality research is surely needed to define the role of the occlusal splints on posture through a combined diagnostic strategy of stabilometric and kinematic evaluation of the spine.

## Figures and Tables

**Table 1:** Search strategy.

*PubMed* (“Temporomandibular Joint Disorders” [MeSH]) AND (“occlusal splints” OR “occlusal splint” OR “stabilization splints” OR “splints” OR “splint”) AND (“posture” OR “postural” OR “postural balance” OR “back” OR “spine” OR “spinal”)
*Scopus* TITLE-ABS-KEY(((“temporomandibular disorders”) AND (“occlusal splints” OR “occlusal splint” OR “stabilization splints” OR “splints” OR “splint”) AND (“posture” OR “postural” OR “postural balance” OR “back” OR “spine” OR “spinal”)))
*Web of Science* ((“temporomandibular disorders”) AND (“occlusal splints” OR “occlusal splint” OR “stabilization splints” OR “splints” OR “splint”) AND (“posture” OR “spinal” OR “postural balance” OR “back” OR “spine” OR “spinal”))

**Table 2:** Reasons for article exclusion by the present systematic review.

Articles excluded after title and abstract screening phase (n = 86) *	
Study design	15 (17.44%)
Not population of interest	23 (26.74%)
Not intervention of interest	42 (48.84%)
Not comparison of interest	45 (52.33%)
Not outcome of interest	51 (59.30%)
*Articles excluded after full-text screening phase (n = * *11* *)*	
Study design	0 (0.00%)
Not population of interest	4 (40.00%)
Not intervention of interest	1 (10.00%)
Not comparison of interest	0 (0.00%)
Not outcome of interest	3 (30.00%)
Full text not available	3 (30.00%)

The exclusion of the articles followed the PICO model defined in the Methods Section. Data are expressed as counts (percentages). * = Papers were excluded also for more than one reason during the title and abstract screening phase and the full-text screening phase.

**Table 3:** Main characteristics of the studies included in the systematic review.

Authors	Journal	Nationality	Design	Pop. (M/F)	Age (Years)	TMD Diagnosis	Intervention	Comparator	Outcome	Time Points	Main Findings
Strini et al., 2009 [53]	Journal of Applied Oral Science	Brazil	Observational	*n* = 20 (1/19)	28.4 ± 8.4	Mixed temporomandibular disorders (TMD) *plus* parafunctional habits (e.g., bruxism)	The upper arch occlusal splint was fabricated programming the mandibular position in centric relation occlusion. The resin rigid occlusal splint was consistent with the technique advocated by Okeson. The patients were instructed to use the splint continuously for 24 h within the first week of treatment and, after this period, to use the splint every night.	N/A	Head position (HP) assessment	Data were collected at baseline (T0), after 1 week (T1), and 1 month (T2).	Regarding physical examination and the patient’s posture, among the values obtained in the HP at the start of treatment, after 1 week and 1 month there were statistically significant differences (*p* < 0.05). In conclusion, the individual’s postural position can suffer biomechanical alterations due to stomatognathic alterations, causing clinically visible changes in dysfunctional individuals and affecting the performance of the involved structures.
Baldini et al., 2012 [54]	Aviation, Space, and Environmental Medicine	Italy	Case Report	*n* = 1 (1/0)	32	Arthrogenous TMD *plus* parafunctional habits (e.g., bruxism)	The lower arch stabilization splint was fabricated programming the mandibular position in centric relation occlusion. The occlusal splint was a resin Michigan rigid splint designed to be worn in flight	N/A	Stabilometric force platform	N/A	The analysis on the force platform showed good postural balance, both with and without the occlusal splint. The clinical gnatho-postural treatment involving a functional diagnostic instrument used on Italian Air Force pilots, protects the masticatory system from dental abrasion and, in this particular case, seemed to improve the pilot’s posture control system as analyzed by the stabilometric platform.
Walczynska-Dragon et al., 2014 [55]	BioMed Research International	Poland	Observational	*n* = 60 (30/30)	33.76 ± 9.1	Mixed TMD *plus* parafunctional habits (e.g., bruxism)	The Sagittal Vertical Extrusion Device (SVED) was a flat-plane appliance which makes contact only with the anterior teeth in the opposing arch. The patients were instructed to use the splints during sleep, but not more than 8–10 h per day	Behavioural therapy	Kinematics of the cervical spine	Data were collected at baseline (T0), after 3 weeks (T1), and 3 months (T2).	The highest improvement was seen during the flexion movement, which, on the 1st examination only in 22% of patients, was within normative values. During the 3rd examination in 70% of patients from treated group, flexion movement conformed to the norm. For the anteflexion movement, the improvement of the results was highly significant (*p* = 0.0006); for the retroflexion movement, the results were improved by a highly significant factor (*p* = 0.0082). In the control group, no significant changes were found. In conclusion, there is a significant association between TMD treatment and reduction in cervical spine pain, as far as improvement of cervical spine mobility.
De Giorgi et al., 2018 [56]	CRANIO^®^: The Journal of Craniomandibular & Sleep Practice	Italy	Randomized controlled trial (RCT)	*n* = 45 (0/45)	41.6 ± 17.3	Arthrogenous TMD	The rigid lower arch occlusal stabilization splint was prepared following the biomechanical models proposed by Ferrario and Sforza, with only posterior contacts (from the second premolar to the second or first permanent molar), without static and dynamic anterior contacts. Patients wore the occlusal splints all night.	No treatment	Rasterstereography	Data were collected at baseline (T0), after 1 month (T1), 3 months (T2), and 6 months (T3).	The evaluation of the cervical arrow at rest position showed a statistically significant difference at T1 between the control group (CG) and the occlusal splint group (SG) (*p* = 0.001). Concerning the kyphotic angle, cervicothoracic inflection point-thoracolumbar inflection point at rest position, a statistically significant difference was found at T1 between CG and SG (*p* = 0.012) and also at T2 between CG and SG (*p* = 0.019). With regard to the lordotic angle, thoracolumbar inflection point-lumbosacral inflection point, a statistically significant difference was found at rest position at T2 between CG and SG (*p* = 0.017). In conclusion, even if some differences were found between the control and the occlusal splint group, the low range of statistical significance made these results not significant from a clinical point of view.
Oliveira et al., 2019 [57]	Clinical and Experimental Dental Research	Brazil	RCT	*n* = 49 (10/39)	39.8 ± 16.3	Mixed TMD	Occlusal stabilization splint *plus* physical therapy. Occlusal splint was performed under the occlusal stability criteria (simultaneous bilateral contacts with absence of interferences in canine and anterior guides). The occlusal splint was used throughout night plus 2 h in the morning and 2 h in the afternoon	Physical therapy	Stabilometric force platform	Data were collected at baseline (T0) and after 12 weeks (T1).	The patients of the test group presented a significant increase in antero-posterior velocity from the center of pressure (COP) with eyes open (*p* = 0.023) and eyes closed (*p* < 0.001). Control group did not present a significant increase in the eyes open (*p* = 0.249) primary outcome only with eyes closed (*p* < 0.046). There was an additional beneficial effect of the use of occlusal splint on the postural balance and guidelines of therapeutic exercises, with a significant increase in antero-posterior velocity of COP of the body with eyes open and closed.
Kang et al., 2020 [58]	Archives of Oral Biology	South Korea	Observational	*n* = 187 (34/153)	35.3 ± 15.4	Mixed TMD *plus* parafunctional habits (e.g., bruxism)/migraine	Upper arch occlusal stabilization splint *plus* physical therapy *plus* behavioral therapy. All patients were instructed to wear the rigid splint every night for at least eight hours per day	N/A	Computerized cephalometric analysis	Data were collected at baseline (T0) and after 6 months (T1).	Using cephalometric analysis, the results demonstrated that controlling the orofacial pain in patients with TMD and migraine showed remarkable improvement in neck pain, head and neck posture, and migraine.
El Zoghbi et al., 2021 [59]	The Journal of Contemporary Dental Practice	Lebanon	Observational	*n* = 47 (0/47)	N/A	Mixed TMD	The upper arch rigid occlusal stabilization splint was worn by patients during night	N/A	Stabilometric force platform	Data were collected at baseline (T0) and after 6 months (T1).	The sway surface area decreased significantly after the occlusal guard placement with closed eyes (*p*= 0.012) but not with eyes open (*p* = 0.169). Likewise, the sway surface area decreased significantly in a dynamic lateral position with closed eyes (*p* = 0.018) and in the anteroposterior dynamic position with open eyes (*p* = 0.031). The mean sway length decreased significantly after the placement of the occlusal splint when participants were in a lateral position with open eyes (*p* = 0.025) and in the anteroposterior position with open eyes (*p*-value, 0.014). In conclusion, in female patients with TMD, the use of an occlusal splint is associated with a postural improvement evaluated by posturo-stabilometric tests.

**Table 4:** Joanna Briggs Institute Critical Appraisal Checklist for Quasi-Experimental Studies (non-randomized experimental studies).

Author and Year	Q1	Q2	Q3	Q4	Q5	Q6	Q7	Q8	Q9
Strini et al., 2009 [53]	N	N/A	N/A	N	Y	Y	Y	N	N
Baldini et al., 2012 [54]	N/A	N/A	N/A	N/A	Y	Y	N	Y	N/A
Walczynska-Dragon et al., 2014 [55]	N	Y	Y	Y	Y	Y	Y	Y	Y
De Giorgi et al., 2018 [56]	Y	Y	Y	Y	Y	Y	Y	Y	Y
Oliveira et al., 2019 [57]	Y	Y	Y	Y	Y	Y	Y	Y	Y
Kang et al., 2020 [58]	N	N/A	N/A	N	Y	Y	Y	Y	Y
El Zoghbi et al., 2021 [59]	N	N/A	N/A	N	Y	Y	N	Y	N

Legend: Q1 = Is it clear in the study what is the ‘cause’ and what is the ‘effect’ (i.e., there is no confusion about which variable comes first)?; Q2 = Were the participants included in any comparisons similar?; Q3 = Were the participants included in any comparisons receiving similar treatment/care, other than the exposure or intervention of interest?; Q4 = Was there a control group?; Q5 = Were there multiple measurements of the outcome both pre and post the intervention/exposure?; Q6 = Was follow up complete and, if not, were differences between groups in terms of their follow up adequately described and analyzed?; Q7 = Were the outcomes of participants included in any comparisons measured in the same way?; Q8 = Were outcomes measured in a reliable way?; Q9 = Was appropriate statistical analysis used?; N = no, Y = yes; N/A = not applicable.

## Data Availability

Not applicable.
